# Are risk predicting models useful for estimating survival of patients with rheumatoid arthritis-associated interstitial lung disease?

**DOI:** 10.1186/s12890-016-0358-2

**Published:** 2017-01-13

**Authors:** Hanna M. Nurmi, Minna K. Purokivi, Miia S. Kärkkäinen, Hannu-Pekka Kettunen, Tuomas A. Selander, Riitta L. Kaarteenaho

**Affiliations:** 1 Center of Medicine and Clinical Research, Division of Respiratory Medicine, Kuopio University Hospital, POB 100, 70029 Kuopio, Finland; 2 Division of Respiratory Medicine, Institute of Clinical Medicine, School of Medicine, Faculty of Health Sciences, University of Eastern Finland, POB 1627, 70211 Kuopio, Finland; 3 Respiratory Medicine, Internal Medicine Research Unit, Medical Research Center Oulu, Oulu University Hospital and University of Oulu, POB 20, 90029 Oulu, Finland; 4 Diagnostic Imaging Center, Division of Radiology, Kuopio University Hospital, POB 100, 70029 Kuopio, Finland; 5 Science Services Center, Kuopio University Hospital, POB 100, 70029 Kuopio, Finland

**Keywords:** Mortality, Rheumatoid arthritis, Interstitial lung disease, RA-ILD, GAP, ILD-GAP, Composite physiologic index

## Abstract

**Background:**

Risk predicting models have been applied in idiopathic pulmonary fibrosis (IPF), but still not validated in patients with rheumatoid arthritis-associated interstitial lung disease (RA-ILD). The purpose of this study was to test the suitability of three prediction models as well as individual lung function and demographic factors for evaluating the prognosis of RA-ILD patients.

**Methods:**

Clinical and radiological data of 59 RA-ILD patients was re-assessed. GAP (gender, age, physiologic variables) and the modified interstitial lung disease (ILD)-GAP as well as the composite physiologic indexes (CPI) were tested for predicting mortality using the goodness-of-fit test and Cox model. Potential predictors of mortality were also sought from single lung function parameters and clinical characteristics.

**Results:**

The median survival was 152 and 61 months in GAP / ILD-GAP stages I and II (*p* = 0.017). Both GAP and ILD-GAP models accurately estimated 1-year, 2-year and 3-year mortality. CPI (*p* = 0.025), GAP (*p* = 0.008) and ILD-GAP (*p* = 0.028) scores, age (*p* = 0.002), baseline diffusion capacity to carbon monoxide (DLCO) (*p* = 0.014) and hospitalization due to respiratory reasons (*p* = 0.039), were significant predictors of mortality in the univariate analysis, whereas forced vital capacity (FVC) was not predictive. CPI score (HR 1.03, *p* = 0.018) and baseline DLCO (HR 0.97, *p* = 0.011) remained significant predictors of mortality after adjusting for age.

**Conclusions:**

GAP and ILD-GAP are applicable for evaluating the risk of death of patients with RA-ILD in a similar manner as in those with IPF. Baseline DLCO and CPI score also predicted survival.

## Background

The course of disease in interstitial lung diseases (ILD), including rheumatoid arthritis-associated interstitial lung disease (RA-ILD), is known to be highly variable. Predicting the survival of an individual patient with ILD is challenging [1]. Several factors have, however, been proposed to predict disease progression and survival i.e. physiological, radiological and histopathological characteristics, as well as demographic variables such as age and gender [2]. Some factors reflecting the severity of the rheumatoid arthritis (RA) have also been associated with worse survival, e.g. baseline pain [3], disease activity score [4] and health-assessment questionnaire score [3, 5].

There are now several indexes which combine single factors into a multifaceted scoring system and these have proved beneficial in estimating prognosis. These models have, however, focused mainly on idiopathic pulmonary fibrosis (IPF) and some of the earliest models were rather cumbersome and therefore never achieved any widespread clinical acceptance [6]. A composite physiologic index (CPI) displayed some important advantages over the older models, since it contained only pulmonary function test (PFT) and gas transfer values but omitted radiological scoring or exercise testing [7]. The subsequently developed GAP model combines gender (G), age (A) and two lung physiology variables (P), i.e. forced vital capacity (FVC) and diffusion capacity to carbon monoxide (DLCO), into a multidimensional index and staging system with three stages (I-III) proposing 1-year mortality of 6, 16 and 39% [8]. This GAP model has also been utilized in the prognosis of other chronic ILDs in addition to IPF. The modified model was named as ILD-GAP, with the assumption that patients with connective tissue disease-related ILDs (CTD-ILD) enjoyed a better survival than those suffering from IPF [9]. The survival of patients with RA-ILD has been shown to be as poor as in IPF patients [10], at least in those cases with usual interstitial pneumonia (UIP) which is the most common subtype in RA-ILD and unlike the situation in the other CTD-ILDs [11]. Thus, since it is mainly UIP-typed, RA-ILD follows a distinctive disease course from the other CTD-ILDs and it remains unclear which of the prognostic indexes, GAP or ILD-GAP, would be better suited for RA-ILD. There are some reports of the benefits of using the CPI score, GAP and ILD-GAP staging systems in patients with IPF and systemic sclerosis-associated ILD [12–14]. However, as far as we are aware, neither CPI nor GAP/ILD-GAP have been previously investigated in patients with RA-ILD, if one excludes the subjects in the original ILD-GAP publication, which did include some RA-ILD patients in their CTD-ILD/idiopathic nonspecific interstitial pneumonia (iNSIP) group of 326 patients.

The aims of this study were to investigate the applicability of CPI, GAP and ILD-GAP scores for predicting the prognosis of the patients with RA-ILD treated in Kuopio University Hospital (KUH), in Eastern Finland, during the years 2000–2014. In addition, we examined the association between individual PFT and demographic factors with the survival of the patients.

## Methods

### Data sources and search

**Fig. 1:**
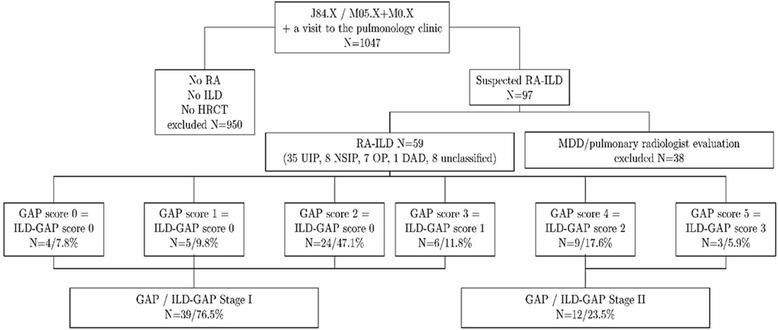
Study protocol. Flowchart of patient enrollment into the study showing the subdivision into the different GAP / ILD-GAP groups. ILD = interstitial lung disease; RA = rheumatoid arthritis; HRCT = high-resolution computed tomography; RA-ILD = rheumatoid arthritis-associated interstitial lung disease; UIP = usual interstitial pneumonia; NSIP = nonspecific interstitial pneumonia; OP = organizing pneumonia; DAD = diffuse alveolar damage; MDD = multidisciplinary discussion; GAP = gender, age, physiologic variables

The study cohort consists of patients treated in the KUH pulmonology in-patient or out-patient clinic between 1.1.2000 and 31.12.2014. The patients were identified from the database of KUH using two International Classification of Diseases (ICD-10) codes, namely J84.X and M05.X/M06.X (Fig. 1). These searches resulted in identification of 1047 patients, and their patient records were evaluated in order to identify those patients suffering from clinically relevant RA-ILD. The search process and clinical characteristics of the patients are thoroughly described in our previous study [15]. Shortly, all patients without a certain diagnosis of RA or without HRCT confirmed ILD were excluded, as were those with mixed CTD-like symptoms. Atypical cases were debated by a multidisciplinary discussion. Finally, 59 radiologically diagnosed RA-ILD patients were identified to be studied in detail and classified adopting the year 2013 IIP classification [16]. The radiological RA-UIP criteria that were applied were those for the diagnosis of IPF [17] when 32 (54.2%) of the patients had a radiological definite UIP pattern [15]. After a multidisciplinary discussion, two additional patients with a slightly upper- or mid-lung predominated distribution where included in the RA-UIP group (35/59.3%), whereas patients with a possible UIP pattern are not included in the UIP group but instead categorized in the unclassified group. In addition to RA-UIP patients, there were eight RA-NSIP (13.6%), seven RA-OP (11.9%), one RA-DAD (1.7%) and eight unclassified patients (13.6%) as previously described [15].

### Gathering of demographic information

Clinical information was gathered from the patient records of KUH, primary health care centers and other hospitals using a specially designed form. Demographic data and the lifelong medication history for RA were gathered comprehensively. The number of hospitalizations was also obtained and further categorized into either mainly respiratory (i.e. infections, suspected drug reactions and suspected acute exacerbations of ILD) or cardiac problems as presented previously [15]. The results of PFT, such as spirometry including FVC and forced expiratory volume (FEV1), as well as DLCO were gathered at baseline and, when available, during the follow-up annually, including also the most recent available results. The reference values of Viljanen were used when assessing PFT results [18].

### Staging systems

Composite physiologic index (CPI) was calculated using the formula [7]: CPI = 91 – (0.65 × DLCO % predicted) – (0.53 × FVC % predicted) + (0.34 × FEV1 % predicted). GAP / ILD-GAP score was calculated by gender, age, FVC % predicted and DLCO % predicted and patients divided to GAP / ILD-GAP stages I and II as previously described [8, 9]. There were no stage III (or IV in ILD-GAP) patients in our study material.

### Statistical analysis

The distribution of the continuous variables was verified with the Shapiro-Wilk test. If there was a normal distribution, the independent *T*-test was used to compare continuous variables, otherwise the Mann-Whitney *U*-test was used. The chi-squared test or Fisher test, when appropriate, was used for comparison of categorical variables. Gender, smoking habits, laboratory results, use of medications, comorbidities, use of oxygen and the numbers of observed deaths were calculated as percentages. Age at the time of RA-ILD diagnosis or death, lung function results and hospitalizations were expressed as mean ± SD. Survival curves were estimated using the Kaplan-Meier method and differences in survival time between GAP / ILD-GAP stages I and II were calculated by the log-rank test. Survival results are expressed as median (95% confidence interval). The observed 1-, 2-, and 3-year mortality rates were calculated and these were supplemented with an estimate of the confidence interval by using the Wilson score. Next, the observed mortality and the risk of death predicted by the GAP / ILD-GAP model were compared using Hosmer-Lemeshow goodness-of fit-test. Finally, Cox regression analysis was used to identify factors that predicted mortality.

*P*-values <0.05 were considered significant. All data was analyzed using IBM Statistics SPSS software, version 21.0.

## Results

### Patient characteristics, lung functions and CPI score

The mean RA duration at the point when ILD was diagnosed was 15.6 ± 12.2 years, ranging from 0 to 52 years. The female–male ratio was 1:1.27. A substantial number (39.7%) of the patients had never smoked. The mean CPI score of all RA-ILD patients was 27.2 ± 14.4 (range 2.4–61.3) (Table 1).

**Table 1:** Pulmonary function test results of the patients with RA-ILD

Variable	Baseline results
FVC	
Normal (>80%)	30 (55.6)
Declined (50–80%)	23 (42.6)
Severely declined (<50%)	1 (1.9)
Normal FEV1 (>80%)	29 (53.7)
Normal FEV1/FVC (>88%)	44 (81.5)
Normal DLCO (>74%)	25 (49.0)
Normal FVC + Normal DLCO	17 (33.3)
Mean FVC	84.76 ± 16.9
Mean FEV1	81.76 ± 16.3
Mean FEV- %	97.59 ± 12.4
Mean DLCO	71.12 ± 18.1
CPI score	27.2 ± 14.4

Data shown as number (%), or mean ± SD. FVC, FEV1 and FEV1/FVC results are missing from five patients. DLCO results are missing from eight patients. Both FVC and DLCO results were available for 51 patients

The detailed data of the lung function test results is shown in Table 1. Over half (30/55.6%) of the patients had a normal FVC at the time of RA-ILD diagnosis. Twenty-five (49%) of the patients had a normal baseline DLCO and furthermore, in 17 patients (33.3%) both FVC and DLCO were normal (Table 1). The clearest decline of all PFT was observed in DLCO, the final mean was 61.1 ± 21.4 (range 13–105).

### GAP and ILD-GAP

There was all the necessary data available from 51 patients to allow the calculation of GAP and ILD-GAP scores. The majority of the subjects i.e. 76.5% (*n* = 39) belonged to stage I with the remaining 23.5% categorized into the stage II group. There were no patients in stage III. The same patients who were categorized as GAP I constituted the ILD-GAP I group and the patients in GAP II group, were also the patients with ILD-GAP II (Fig. 1). GAP / ILD-GAP I and II differed significantly with respect to several clinical findings and lung function e.g. age (*p* = 0.024), gender (*p* < 0.001), smoking status (*p* = 0.033), baseline FVC (*p* < 0.001), FEV1 (*p* = 0.013) and DLCO (*p* < 0.001) (Table 2). The use of methotrexate was also more common in stage I patients than in their stage II counterparts (64.1% vs. 33.3%), although this finding did not reach statistical significance (*p* = 0.060). No statistically significant differences were observed in RA serology or comorbidities. The mean CPI score was 22.4 ± 12.2 in GAP / ILD-GAP I and 42.8 ± 9.3 in stage II (*p* < 0.001). Patients with the UIP pattern in HRCT (RA-UIP) divided almost equally in both stages (64% in stage I, 50% in stage II, *p* = 0.502).

**Table 2:** Baseline characteristics of the patients with RA-ILD

	GAP / ILD-GAP stage I (*n* = 39/76.5%)	GAP/ILD-GAP stage II (*n* = 12/23.5%)	*P*-value
Age (y)	63.4 ± 11.6	71.5 ± 5.5	0.024
Age at death (y)	72.6 ± 9.9	76.6 ± 5.6	0.266
Male sex	16 (41.0)	12 (100.0)	<0.001
UIP pattern	25 (64.1)	6 (50.0)	0.502
Smoking*			
Never	19 (48.7)	1 (9.1)	0.033^a^
Ex-smoker	15 (38.5)	7 (63.6)	
Current smoker	5 (12.8)	3 (27.3)	
Serology			
Positive RF**	29 (78.4)	11 (91.7)	0.420^a^
Positive ANA***	4 (14.3)	2 (22.2)	0.620^a^
Medications			
Steroids	36 (92.3)	10 (83.3)	0.580^a^
MTX	25 (64.1)	4 (33.3)	0.060
Biological drugs	11 (28.2)	1 (8.3)	0.250^a^
Lung functions			
FVC % pred	89.8 ± 15.8	72.6 ± 8.5	<0.001
FEV1 % pred	85.1 ± 16.3	72.3 ± 8.9	0.013
DLCO % pred	76.8 ± 15.6	52.8 ± 12.7	<0.001
RA duration (y)	15.7 ± 10.6	14.8 ± 14.4	0.808
CPI points	22.4 ± 12.2	42.8 ± 9.3	<0.001

For eight patients there was no lung function data and therefore the GAP / ILD-GAP score could not be calculated

*RF* rheumatoid factor, *ANA* antinuclear antibodies, *MTX* methotrexate, *FVC* forced vital capacity, DLCO diffusing capacity of the lung for carbon monoxide, *% pred* percentage of the predicted value, *CPI* composite physiologic index

*Data missing from one stage II patient

**Data missing from two stage I patients with positive anti-cyclic citrullinated peptide antibodies

***Data missing from 14 patients (11 stage I, 3 stage II)

^a^Fisher test

### The follow-up outcomes

No statistically significant differences were observed between GAP / ILD-GAP I and II with regard to hospital admissions either due to respiratory or cardiologic reasons (Table 3). The use of oxygen was also similar in both groups (*p* = 1.000). Eighteen patients (46.2%) died due to any cause in the GAP / ILD-GAP stage I whereas there were 9 deceased patients (75.0%) in the stage II group (*p* = 0.080). The observed cumulative mortality rates at 1, 2 and 3 years were 7.0, 16.7 and 22.6%, respectively. The observed 1-year, 2-year or 3-year mortality did not differ significantly according to GAP / ILD-GAP stage.

**Table 3:** Course of disease and survival of the patients with RA-ILD

	GAP / ILD-GAP stage I (*n* = 39/76.5%)	GAP / ILD-GAP stage II (*n* = 12/23.5%)	*P*-value
Number of deaths	18 (46.2)	9 (75.0)	0.080
Hospitalization due to respiratory illness	1.0 ± 1.4 (0–5)	1.6 ± 3.1 (0–11)	0.343
Hospitalization due to cardiac illness	0.5 ± 1.0 (0–5)	1.0 ± 1.8 (0–4)	0.366
Use of Oxygen	6 (15.4)	1 (8.3)	1.000^a^
Median survival	152.0 (93.0–211.0)	61.0 (25.2–96.8)	0.017
Observed 1-y deaths*	0 (0.0)	1 (8.3)	0.245
Observed 2-y deaths**	5 (14.3)	1 (9.1)	1.000
Observed 3-y deaths***	6 (17.6)	3 (27.3)	0.666

Categorical variables are compared using the Fisher test when marked ^a^, otherwise *χ* ^2^-test. Hospitalizations are compared using the Mann-Whitney *U*-test

*Data missing from two stage I patients

**Data missing from four stage I patients and one Stage II patient

*** Data missing from five Stage I patients and one Stage II patient

### Survival and validation of the GAP and ILD-GAP models

**Fig. 2:**
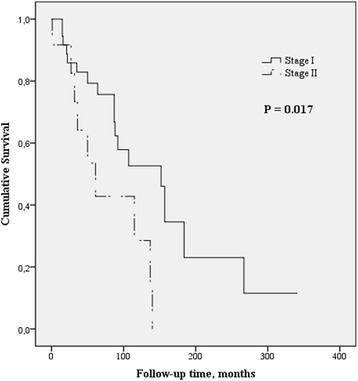
Comparison of the survival curves of the RA-ILD patients categorized into either GAP / ILD-GAP stage I or II. The survival was significantly worse in GAP / ILD-GAP stage II (*p* = 0.017, Log Rank)

The median survival was 152 months in stage I but only 61 months in stage II (*p* = 0.017) (Fig. 2). There were no apparent differences in the observed and predicted risk of death (Table 4). Both prediction models fitted the Wilson score confidence interval of the observed mortality.

**Table 4:** Predicted and observed cumulative mortality of the patients with RA-ILD

GAP/ILD-GAP stage	Observed	Predicted by GAP index and staging system	Predicted by ILD-GAP
1-Y mortality			
Stage I	0.0 (0.0–9.4)	5.6	3.1
Stage II	8.3 (1.5–35.4)	16.2	8.8
2-Y mortality			
Stage I	14.3 (6.3–29.4)	10.9	6.6
Stage II	9.1 (1.6–37.7)	29.9	18.0
3-Y mortality			
Stage I	17.6 (8.3–33.5)	16.3	10.2
Stage II	27.3 (9.7–56.6)	42.1	26.9

% (95% CI calculated by Wilson score)

**Fig. 3:**
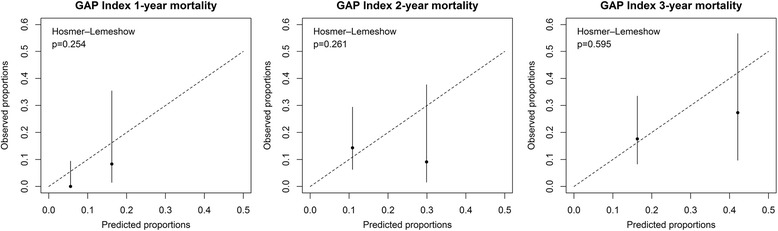
The Hosmer-Lemeshow statistic tests show that the predicted and observed risks do not differ significantly (*p* > 0.05). The *x-axis* shows the 1-y, 2-y and 3-y risk of mortality as predicted by the GAP staging system and the *y-axis* shows the observed risk. In every figure, stage I is on the left side and stage II on the right side. The *vertical lines* represent the confidence interval of the observed mortality rate

**Fig. 4:**
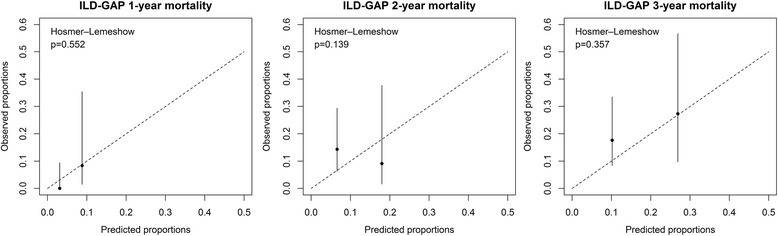
The Hosmer-Lemeshow statistic tests show that the predicted and observed risks do not differ significantly (*p* > 0.05). The *x-axis* shows the 1-y, 2-y and 3-y risk of mortality as predicted by the ILD-GAP staging system and the *y-axis* shows the observed risk. In every figure, stage I is on the left side and stage II on the right side. The *vertical lines* represent the confidence interval of the observed mortality rate

The observed mortality and the risk of death predicted by these models were compared using the Hosmer-Lemeshow goodness-of-fit test (Figs. 3 and 4). Both GAP and ILD-GAP indexes predicted 1-year, 2-year and 3-year mortality accurately (all p-values were > 0.05). The ILD-GAP index was more accurate at predicting 1-year mortality (*p* = 0.552) than the GAP index (*p* = 0.254). However, the GAP index was slightly more accurate at predicting 2-year (*p* = 0.261) and 3-year (*p* = 0.595) mortality than the ILD-GAP index (2-year *p* = 0.139, 3-year *p* = 0.357).

### Predictors of mortality

GAP and ILD-GAP indexes, as well as the CPI score were all significant predictors of mortality when assessed with the univariate Cox model. The hazard ratio (HR) of GAP was 1.56 (95% CI: 1.15–2.11; *p* = 0.004), that of ILD-GAP 1.51 (95% CI: 1.05–2.18; *p* = 0.026) and of CPI 1.03 (95% CI 1.01–1.06; *p* = 0.015) (Table 5).

**Table 5:** Prognostic factors for survival in patients with RA-ILD using a univariate Cox model

	Hazard ratio	95% CI	*P*- value
Age at diagnosis	1.06	1.02–1.10	0.002
Male sex	1.49	0.73–3.05	NS
Smoking	0.83	0.41–1.67	NS
FVC % pred	0.98	0.96–1.01	NS
DLCO % pred	0.98	0.96–1.00	0.014
RA duration	0.99	0.96–1.03	NS
UIP pattern in HRCT	0.77	0.36–1.64	NS
Positive RF	0.69	0.24–1.98	NS
MTX	1.20	0.59–2.42	NS
Use of oxygen	1.74	0.74–4.09	NS
Resp. hospitalization	1.12	1.01–1.26	0.039
Card. hospitalization	1.13	0.87–1.46	NS
CPI- points	1.03	1.01–1.06	0.015
GAP score	1.56	1.15–2.11	0.004
ILD-GAP score	1.51	1.05–2.18	0.026

Age at diagnosis (HR 1.06, 95% CI 1.02–1.10, *p* = 0.002), baseline DLCO (HR 0.98, 95% CI 0.96–1.00, *p* = 0.014) and hospitalization due to respiratory reasons (HR 1.12, 1.01–1.26, *p* = 0.039) were also significant predictors of mortality in the univariate model, but neither FVC nor hospitalization due to cardiologic reasons was predictive. The UIP pattern was not an independent risk factor in this cohort, neither was smoking nor male gender. The use of either methotrexate or oxygen did not reach statistical significance as risk factors for death (Table 5).

### Age adjusted predictors of mortality

After adjusting for age, CPI score and baseline DLCO remained as significant predictors of mortality. For every increased CPI point, the mortality risk increased by 3% (HR 1.03, 95% CI 1.01–1.06, *p* = 0.014) and for every increased DLCO level, the risk of death diminished by 3% (HR 0.97, 95% CI 0.95–0.99, *p* = 0.011). The rest of the factors that were detected in the univariate Cox model lost their statistical significance after adjustment for age (Table 6).

**Table 6:** Prognostic factors for survival after adjustment for age

	Hazard ratio	95% CI	*P*- value
DLCO % pred	0.97	0.95–0.99	0.011
Resp. hospitalization	1.11	0.99–1.26	0.084
CPI- points	1.03	1.01–1.06	0.014
GAP score	1.37	0.96–1.94	0.083
ILD-GAP score	1.32	0.90–1.95	0.158

## Discussion

In this present study, we applied the GAP and the ILD-GAP scores in a cohort consisting of 59 patients with RA-ILD subdivided into GAP / ILD-GAP stages I and II. Both GAP systems showed significant differences in age, gender, FVC, FEV1, DLCO and CPI-score, which is understandable since GAP / ILD-GAP are mainly composed of the above-mentioned components. The median survival of the patients categorized into GAP / ILD-GAP II groups was significantly shorter than those in the GAP / ILD-GAP I group. The CPI score was an independent predictor of mortality similarly as GAP / ILD-GAP scores, age, baseline DLCO and hospitalization due respiratory reasons. However, after adjustment for age, only the CPI score and DLCO remained as statistically significant predictors. In addition to the Cox model, the applicability of GAP and ILD-GAP was tested using two different statistical methods. Both the GAP and the ILD-GAP methods provided relatively good estimates of mortality. Interestingly, the GAP index was more accurate at predicting 2-year and 3-year mortality, whereas ILD-GAP predicted 1-year mortality more precisely.

To our knowledge, only a few previous studies have investigated GAP or ILD-GAP scores in patients with CTD-ILD but some analyses of IPF have been published. In Korean IPF patients, the GAP score produced accurate 1-year, but not 3-year, mortality estimates [13]. In another study of IPF patients, the GAP staging was found to be useful for evaluating the IPF severity, revealing statistically significant differences in survival in different GAP stages [12]. On the other hand, the ILD-GAP index displayed poor applicability for the predicted 1-year mortality in systemic sclerosis-associated ILD patients [14].

In this study, the observed 1-year mortality was 0 in stage I and 8.3% in stage II patients. Predicted 1-year mortality using the ILD-GAP was 3.1 and 8.8% in stages I and II, respectively. Thus, the accuracy of ILD-GAP was good at predicting 1-year mortality but the observed 2-year mortality in stage I patients was much higher than predicted by the ILD-GAP model i.e. the GAP model was more accurate at that time point. The ILD-GAP prediction also underestimated the 3-year mortality of stage I patients, which was observed to be 17.6 and therefore was even slightly higher than the value predicted by GAP. Both of the indexes, however, fitted within the confidence interval of the observed mortality. Since the accuracy of GAP and ILD-GAP in predicting annual mortality in our study was variable at different points, it remains unclear whether the GAP or ILD-GAP index is better suited in predicting mortality of patients with RA-ILD. The ILD-GAP was originally developed in a study protocol including all kinds of ILDs without taking into account the fact that the prognosis and course of disease is variable in the different types CTD-ILDs [19, 20]. In some earlier studies, the survival of RA-ILD patients has been reported as being as poor as in IPF [3, 21], whereas that of other types of CTD-ILD has appeared to be better [10, 19, 22]. Furthermore, various radiological or histological patterns in certain CTD may behave differently, e.g. patients with RA-UIP have been shown to have a shorter survival than those with other CTD-ILDs [23, 24]. Therefore, it can be debated whether the ILD-GAP, which is merely a simple subtraction from the GAP score assuming a better survival in CTD-ILDs, is valid in all CTD-ILDs.

The significance of PFT has been widely recognized when evaluating ILD severity and the risk of death. In fibrotic subtypes of IIPs, it has been postulated that pulmonary physiology is an even stronger predictor of survival than the histopathologic pattern [25] and that in patients with IPF, changes in FVC % predicted and DLCO % predicted have been shown to associate with mortality [26, 27]. Moreover, a prospective follow-up study of 29 RA-ILD patients demonstrated that in over 30% of cases, a degree of radiological progression was observed, and this progression was strongly associated with a reduced DLCO [28].

In a recent retrospective study of 48 biopsy-confirmed RA-ILD patients, the baseline DLCO was detected as an important risk factor for death in a univariate model similarly as found here [29]. In that particular study, however, DLCO lost its statistical significance in the multivariate model, when only age and the presence of fibrosis remained significant [29]. Another study of 82 RA-ILD patients diagnosed without biopsy found that baseline DLCO was associated with survival in the bivariate analysis, and DLCO remained statistically significant also in the multivariate analysis [30]. In a very recent study, a relatively large cohort of 137 RA-ILD patients was retrospectively evaluated, with univariate, multivariate and also longitudinal methods being used to analyze the predictors of mortality [31]. In that study a baseline DLCO value of 10% lower than the mean value and DLCO decline of 10% or more at any time after baseline were identified as significant predictors of mortality [31]. Furthermore, in the study of Song et al. [32] which examined 84 RA-UIP patients, the hazard ratio of baseline DLCO did not reach statistical significance, but the change of DLCO was significant in both univariate and multivariate models. Unfortunately, we were not able to investigate the change in DLCO over time because of missing follow-up data due to the retrospective nature of our study protocol. In addition, multivariate models could not be applied because of the small number of patients in our study. However, we observed that the significant positive result of DLCO in univariate analysis remained after adjusting for age. Overall, the results of DLCO in our study support the previous findings of the suitability of DLCO in the disease severity evaluation of RA-ILD.

Baseline FVC was not found to be an independent predictor of mortality in our study, a finding which is at odds with some previous studies. A recent study showed that the lower baseline FVC (10% or more under mean value) and a 10% decline in FVC were both associated with an increased death hazard in various multivariate models [31]. Furthermore, another investigation demonstrated that the baseline FVC, as well as the FVC change over time, were significant predictors of mortality in patients with RA-UIP [32]. There may be two possible explanations why the significance of FVC in our study differs from these other publications. Firstly, in our study, the mean baseline FVC was relatively high, i.e. 84.8, being within the normal limits in the majority of the patients whereas the corresponding value in the study of Solomon et al. was 69.3, and that from Song et al. was 75.1 [31, 32]. Our finding refers that the patients had been diagnosed earlier with more preserved lung functions. Secondly, our study includes 59 patients, thus being relatively small, compared to those other studies of 84 and 137 patients. On the other hand, the results of the study of Kim et al., which included 84 patients with RA-ILD who had lower mean baseline of FVC values than in our study (66 ± 25 in RA-UIP, 70 ± 20 in non-UIP) did not actually find FVC to be a predictor of death, i.e. similar to our results [23]. Even though FVC alone was not a strong predictor of mortality in our study, it is one factor included in CPI and GAP / ILD-GAP scores, all of which showed significant positive results in our univariate analyses. Our finding supports that the use of multifaceted scoring systems for evaluating the prognosis of the patients with RA-ILD may be beneficial.

## Conclusions

In conclusion, GAP, ILD-GAP and CPI were all functional when predicting survival of patients with RA-ILD. In addition, baseline DLCO was associated with length of remaining lifetime. In clinical practice, reliable methods are needed for evaluating the progression of the disease and predicting an individual’s life expectancy and predictive scoring systems could be helpful in everyday work and in patient counselling. Hopefully in the future, more disease-specific methods can be developed and validated, although this would require additional multicenter studies.

## Abbreviations

ANA: Antinuclear antibodies
CI: Confidence interval
CPI: Composite physiologic index
CTD: Connective tissue diseases
DAD: Diffuse alveolar damage
DLCO: Diffusion capacity to carbon monoxide
FEV1: Forced expiratory volume
FVC: Forced vital capacity
GAP: Gender, age and physiological variables
HR: Hazard ratio
HRCT: High-resolution computed tomography
IIP: Idiopathic interstitial pneumonias
ILD: Interstitial lung disease
ILD-GAP: Interstitial lung disease – gender, age, physiology
iNSIP: Idiopathic nonspecific interstitial pneumonia
IPF: Idiopathic pulmonary fibrosis
KUH: Kuopio University Hospital
MDD: Multidisciplinary discussion
NSIP: Nonspecific interstitial pneumonia
OP: Organizing pneumonia
PFT: Pulmonary function test
RA: Rheumatoid arthritis
RA-ILD: Rheumatoid arthritis-associated interstitial pneumonia
RA-UIP: Rheumatoid arthritis-associated usual interstitial pneumonia
RF: Rheumatoid factor
SD: Standard deviation
UIP: Usual interstitial pneumonia
